# Skeletal Muscle Dystrophy mutant of lamin A alters the structure and dynamics of the Ig fold domain

**DOI:** 10.1038/s41598-018-32227-2

**Published:** 2018-09-14

**Authors:** Subarna Dutta, Jitendra K. Das, Lakshmi Maganti, Maitree Bhattacharyya, Dhananjay Bhattacharyya, Sujoy Mukherjee, Kaushik Sengupta

**Affiliations:** 1 Biophysics & Structural Genomics Division, Saha Institute of Nuclear Physics, Homi Bhabha National Institute, 1/AF Bidhan nagar, Kolkata, 700064 West Bengal India; 2 Structural Biology & Bio-Informatics Division, CSIR Indian Institute of Chemical Biology, 4, Raja S. C. Mullick Road, Kolkata, 700032 West Bengal India; 3 Computational Science Division, Saha Institute of Nuclear Physics, Homi Bhabha National Institute, 1/AF Bidhan nagar, Kolkata, 700064 West Bengal India; 4 Department of Biochemistry, University of Calcutta, 35 Ballygunge Circular Road, Kolkata, 700019 West Bengal India

## Abstract

Mutations in the different domains of A-type lamin proteins cause a diverse plethora of diseases collectively termed as laminopathies which can affect multiple organs. Ig fold is one such domain of lamin A which is implicated in numerous nuclear interactions wherein the mutations lead to different laminopathies. W514R is one such mutation in the Ig fold which leads to severe phenotypes in Skeletal Muscle Dystrophy (SMD) which is a class of laminopathies. In this report, we elucidated gross alterations in structure and dynamics at the level of individual amino acids. These studies indicate altered conformational features of residues in the close vicinity of W514. Imaging of mammalian cells transfected with the mutant have shown distinct perturbation of the nuclear meshwork with concomitant alteration in nuclear interactions as a result of increased oligomerization of Ig W514R. Hence, this novel approach of amalgamating theoretical and experimental procedures to predict the severity of a mutant in the context of laminopathies could be extended for numerous lamin A mutants.

## Introduction

Lamins are type V intermediate filament protein of nuclear origin. These proteins are present in all metazoan cells^1^ as a network of 3.5 nm thick filaments^2^ underneath the inner nuclear membrane^3^.This protein mesh or lamina of approximately 14 nm thickness^1^ provides adequate mechanical rigidity to the nucleus^4–7^. Although the lamina is not uniformly thick throughout the periphery of the nucleus, it provides a basic framework for chromosome tethering and modulates vital processes like DNA replication, DNA damage repair and transcription in conjugation with other nuclear proteins^8^. A- and B-type lamins constitute the mammalian lamin pool. Lamins A/C being encoded by LMNA gene as alternate splice variants are weakly expressed in embryonic stem cells^9^ but significantly up regulated at the onset and during the process of differentiation^10–12^. On the other hand, major B-type lamins B1 & B2 are expressed from LMNB1 &LMNB2, respectively, right from embryonic stage^13^. Minor isoforms include AΔ10 and lamin B3 which are expressed in a tissue specific manner^14,15^. The observation that B-type lamins appear early in the process of embryogenesis finds an explanation from its stiffness parameter. Lamin B1 has been shown to form stress resistant and resilient fibres which are being able to withstand the mechanical strain upto a certain limit. On the other hand lamin A/C null embryonic stem cells are highly plastic which stiffens with differentiation. Similar situation is encountered in the process of differentiation of myoblast to myotube. Based on these observations and our previous data on viscoelastic behaviour of lamin A we argued that lamin A filaments top up and reinforce previously existing B-type filaments thereby imparting adequate rigidity to the nucleus in response to growing mechanical strain^16^.

Lamins possess a long α-helical coiled-coil forming rod domain flanked by an unstructured N-terminal head and a C-terminal tail^17^. Though grossly unstructured, the C-terminal tail has many essential characteristic motifs – a Nuclear Localization Signal (NLS) followed by an immunoglobulin Ig fold domain and a CAAX box at the extreme C-terminus harbouring the site for farnesylation^18^. The structure of the Ig fold domain has been solved by both NMR and X-Ray crystallography^19,20^ and is reminiscent of the s-type immunoglobulin^21,22^. It is a β sandwich structure of two β-sheets containing 5 β-strands and 4 β-strands respectively interconnected by short loops and is referred to as L-subtype of immunoglobulin^20^. In retrospect, the C-terminal domain of lamin A/C has been ascribed to be a site of interaction with many nuclear proteins including LAP2α, emerin, PCNA, SREBP1^23–25^. The interaction of Ig fold with emerin and PCNA was conclusively proven earlier^26,27^. Furthermore strong evidence from earlier studies reported the binding of NPC proteins Nup88 and Nup153 to the Ig fold domain^28,29^. Recently a SUMO-interacting site has been uncovered in Ig fold of lamin A/C which might be important in mediating protein-protein interaction with SUMOylated substrates^30^. This justifies the objective of studying the structural and temporal perturbations of the Ig fold domain in great details.

Since the past two decades there has been an upsurge in the research pertaining to lamin A/C and the mutants thereof due to the ongoing discovery of an overwhelming number of mutations which produce at least 13 different diseases collectively called laminopathies (http://www.umd.be/LMNA/). Based on the type of tissues being affected, laminopathies can be broadly classified into muscular dystrophies, lipodystrophies, neuropathies and premature aging syndrome^31,32^. Although the molecular mechanisms for these diseases involving lamin A/C are poorly understood, contributing factors seem to be impaired mechanosensitivity due to change in nuclear elasticity^16,33^, altered chromatin maintenance^34^ and overall modulation of nuclear signalling pathways^35^. Several hypotheses have been in vogue to explain the pathophysiology of these diseases. The structural hypothesis points to the fact that the nuclear lamina and hence the nucleus becomes structurally weak due to the lamin A/C mutations leading to misshapen/fragile nuclei which are unable to resist excessive mechanical strain as is encountered in skeletal muscles. The second hypothesis pertains to differential gene regulation during differentiation of tissues particularly muscles as a sequel to mutations in LMNA gene. This might occur due to aberrant interactions of different transcription factors with lamin A/C. It has been shown that myoblasts with low levels of lamin A/C show a reduction in the expression profiles of MyoD, desmin and cadherin^36^. Over 400 missense mutations have been isolated in lamin A/C that are spread throughout the entire protein stretch, of which nearly 15% are concentrated in the Ig fold domain. Approximately 45% of these Ig fold mutations (~27) are associated with Skeletal Muscle Dystrophy (SMD). Moreover these SMD mutants map in the region of the Ig fold harbouring distinct negative charge^37^ thereby leading to a gross destabilization of the Ig fold as reported by Krimm *et al*. for the mutants R453W, R482W and R482Q^19^.

In this work we have elucidated the detailed structural perturbations of Ig W514R mutant along with the dynamics. Although this mutant leads to severe symptoms in patients and has been studied in drosophila myogenesis defects, the residue wise structural changes in the domains are not delineated by transverse /longitudinal relaxation rates and also MD simulation^38^. The aim of this work is to analyze, the structural fluctuations of the Ig fold domain due to this mutation and corresponding changes in the dynamics of individual residues. We have thereby provided essential details of the structure by fluorescence, CD spectroscopy and high resolution NMR spectroscopy. Furthermore, we have used molecular dynamics simulations to elucidate residue wise changes in the dynamics of the mutant protein. To stress upon the biological effects of this mutation on the nucleus, we have shown the perturbation of the nuclear lamina as a consequence of this mutation along with changes in interaction profile with other nuclear proteins.

## Results

### Expression and characterization of Ig W514R

**Figure 1:**
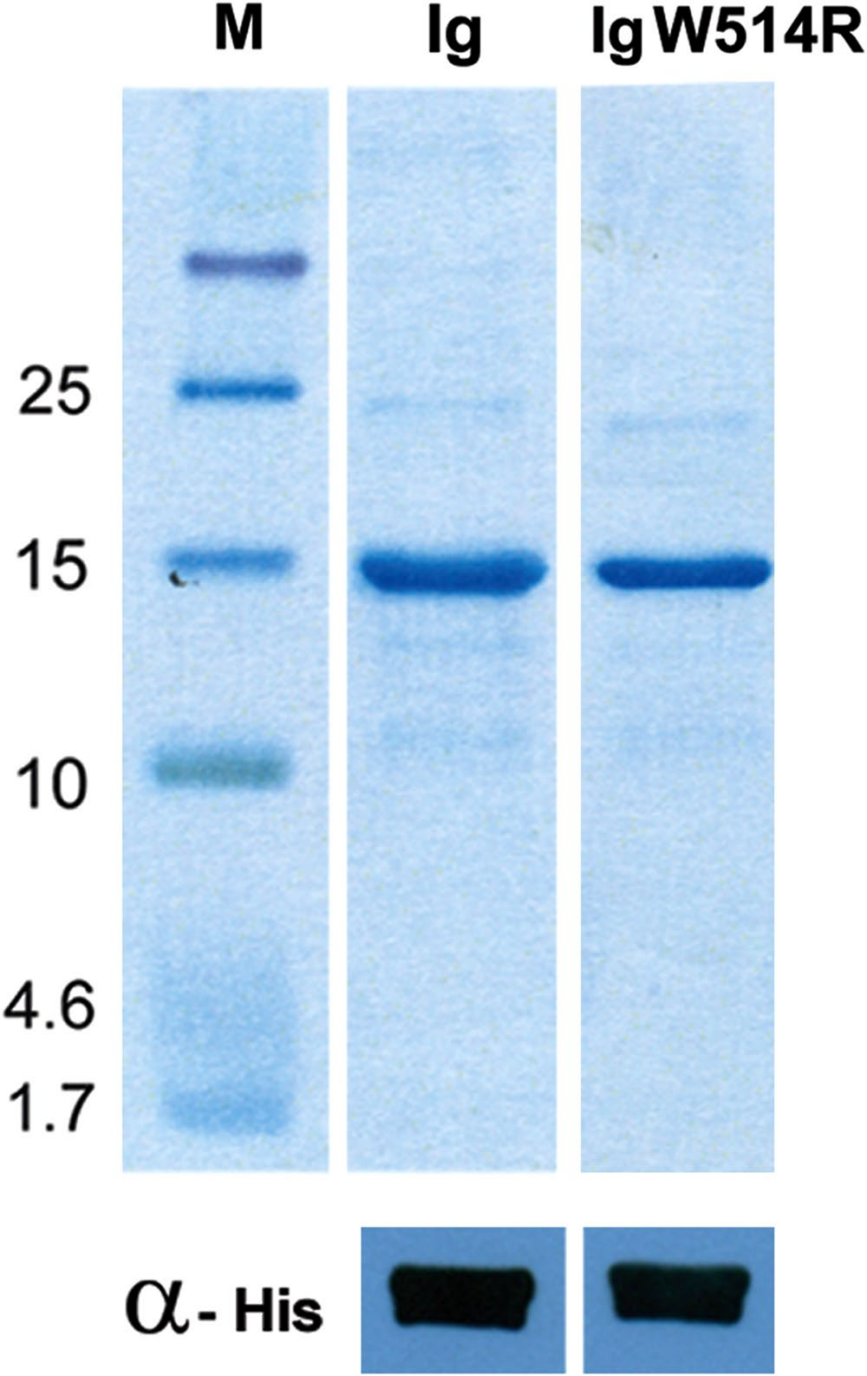
Overexpression profile of Ig proteins: (**a**) Over expressed Ig and Ig W514R purified to near homogeneity have been analysed on 4–20% gradient gel in the top panel. The bottom panel shows characterization of the proteins by anti-His antibody. The strips shown in the gel/blot are cropped from the original gels/blot as shown in Supplementary Fig. S7.

In order to describe our results with the purified proteins, we hereby adopt the convention of referring to the native and mutant proteins as Ig and Ig W514R respectively. These proteins were robustly expressed in a heterologous manner in *E. coli* strain BL21 (DE3) pLysS and purified to homogeneity (Fig. 1) by affinity chromatography on His-Trap column. Ig could be obtained up to 6–7 mg/ml (0.46 mM) concentration whereas Ig W514Rprecipitated at that concentration and remained stable at 3–4 mg/ml (0.23 mM) in PB. The purity of the proteins was further confirmed by western blot using antibody recognizing the domain as epitope.

**Figure 2:**
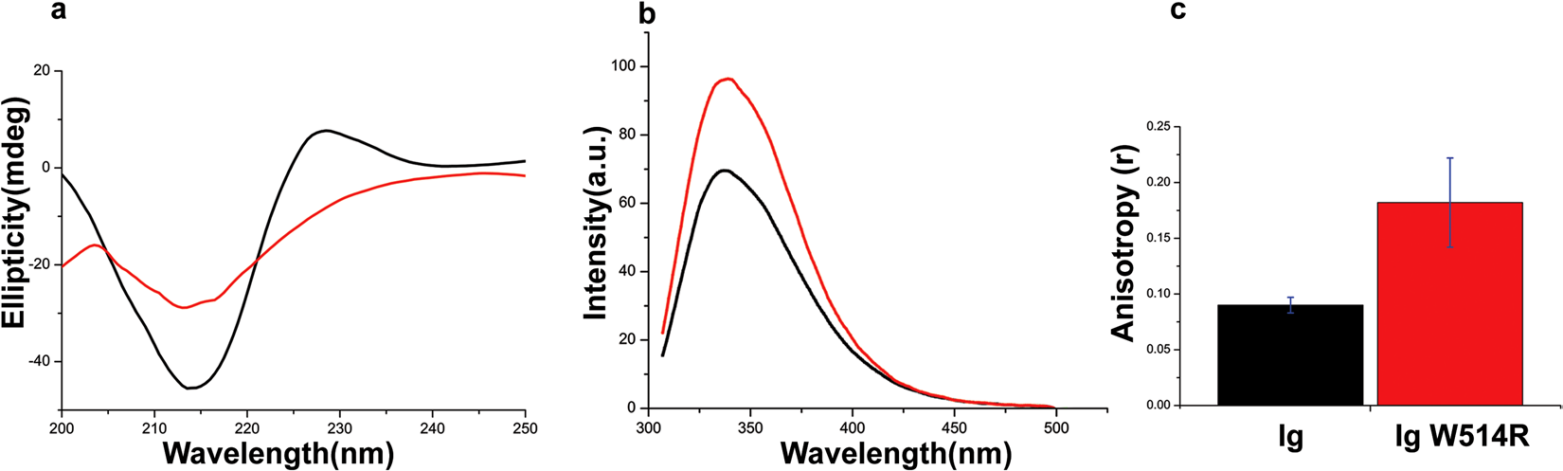
Ig W514R alters the secondary and tertiary structure: (**a**) Far UV CD spectra of Ig (black)and Ig W514R (red) at 25 °C. (**b**) Steady state fluorescence emission spectra (λex = 295 nm) of Ig (black) and Ig W514R (red). (**c**)Fluorescence anisotropy values of Ig and Ig W514R are plotted as bar graphs incorporating the standard errors. Fluorescence anisotropy values of Ig and Ig W514R are plotted as bar graphs incorporating the standard errors.

Subsequently, we recorded the CD spectra at concentration of 10 µM (Fig. 2a). The mutant showed a marked deviation in the secondary structure which consists of a β-sandwich structure comprising of 9 β-strands. The π-π* transition (228 nm) and n-π* (213 nm) transition got significantly altered which could possibly represent a perturbation in the β-sheet(s). This is similar to the previous results obtained by Dialynas *et al*.^38^ which is most likely to influence the gross tertiary structure of the β-sandwich. We used steady state fluorescence spectroscopy to probe any perturbation in the tertiary structure at the concentration of 10 µM for each of Ig and Ig W514R. From the spectra (Fig. 2b**)** we can conclude that there is an alteration of the Trp (W) microenvironment in the mutant as a sequel to the mutation. Interestingly, all the 4 Trp residues lie in the Ig fold domain of lamin A^39^. The mutant registered ~43% increase in quantum yield compared to the wild type concomitant with blue shifted emission maxima by 2 nm. This is only possible if the Trp microenvironment remains more protected as a hydrophobic core. It can be conjectured that increased homooligomerization amongst the Ig W514R monomers promotes the Trp residues to remain deeply buried and precludes any possibility of solvent induced quenching thereby promoting blue shifted maxima with increased quantum yield. We also analyzed the steady state fluorescence anisotropy of Ig and Ig W514R at same concentrations, which showed that the anisotropy (r) was more than 2, folds that of Ig (Fig. 2c). In other words, Ig W514R might have undergone a transition to a more voluminous structure, presumably due to increased oligomerization, hence restricting its rotational tumbling motion in the buffer medium.

### NMR reveals difference in structure and dynamics between Ig and Ig W514R

**Figure 3:**
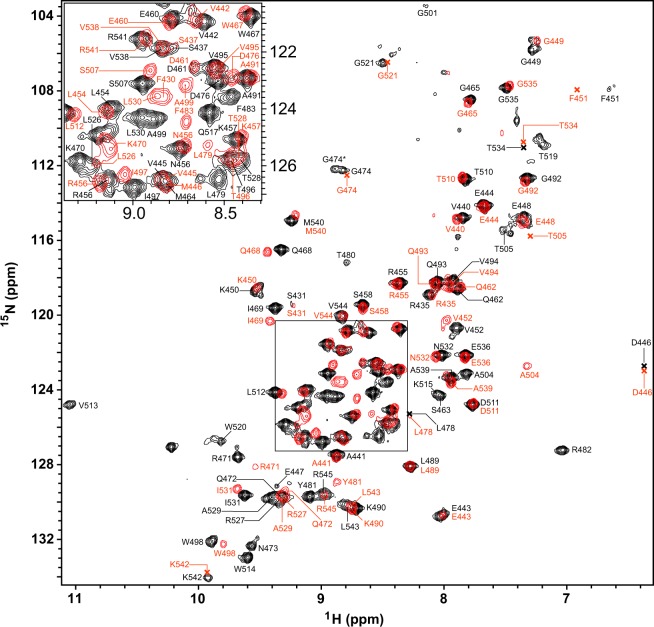
^15^N-^1^H HSQC spectra of Ig fold domain, wild type (black) and mutant W514R (Red), acquired at 14.2 T external magnetic field with the backbone resonance assignments.

**Figure 4:**
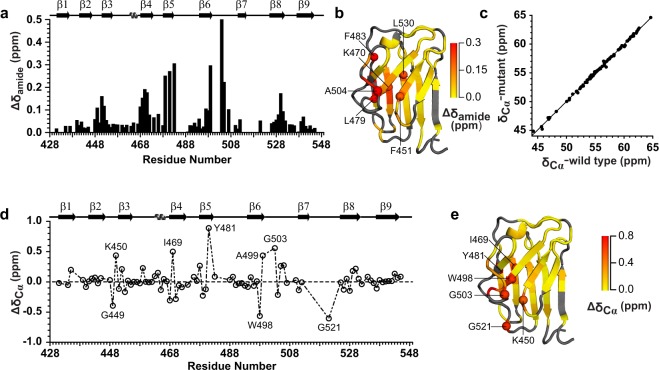
NMR chemical shift comparison between wild type Ig and mutant Ig W514R: (**a**) Residue specific plot of amide chemical shift changes between the wild type and the mutant Ig W514R, (**b**) Values of amide chemical shift changes mapped on the NMR structure (PDB: 1IVT)^12^. Some representative residues with relatively higher value of backbone amide chemical shift change are shown in spheres. (**c**) Correlation curve between the ^13^C^α^ chemical shift of wild type *vs*. mutant Ig W514R (r^2^ = 0.998). (**d**) Plot of residue specific ^13^C^α^ chemical shift differences between the wild type and the mutant Ig W514R, (**e**) ^13^C^α^ chemical shift differences as mapped on the NMR structure. Some of the residues showing relatively significant differences between the wild type and the mutant are shown as spheres in the NMR structures.

In order to have a residue specific understanding of structure and conformational dynamics of the wild type and the mutant Ig W514R, we acquired two dimensional {^15^N-^1^H} heteronuclear single quantum coherence (HSQC) spectra of both the wild type and the mutant (Fig. 3), wherein the peaks indicate a correlation between amide ^1^H and ^15^N spins. The extent of unobservable NMR peaks was significantly higher for the mutant than the wild type lamin Ig. In retrospect, the disappearance of peaks due to excessive line broadening is indicative of molecular motions due to chemical exchange of residues between two or more conformational states. This corroborates with our earlier observation, where we concluded the increased tendency of the mutant Ig W514R to oligomerize. Not surprisingly, majority of the missing resonances, including residues K486-T488, A500-G503 and V513-W520, appear in unstructured loop regions of the protein near the site of the mutation that can be reasonably expected to exhibit increased conformational dynamics. The overall chemical shift dispersion of resonances of the HSQC spectra (Fig. 3) indicated that both of the proteins have structured conformations. However, differences in the spectra suggested that their structures were different. This was further investigated by calculating the change in various {^1^H,^15^N} chemical shifts between the wild type and the mutant proteins (Fig. 4a) since chemical shift is dictated by the electronic environment surrounding the amide region and changes in amide resonances would indicate changes in the tertiary structure of the protein. Moreover, backbone ^13^C^α^ chemical shift is an accurate reporter of secondary structure whereas changes in backbone {^1^H,^15^N}amide frequencies indicate perturbation to the tertiary fold^40^. The overall change in the amide chemical shift between the wild type and the mutant was calculated using the expression

$${{\rm{\Delta }}{\rm{\delta }}}_{{\rm{amide}}}=\sqrt{{{({\rm{\Delta }}{\rm{\delta }}}_{{\rm{HN}}})}^{{\rm{2}}}{+({\rm{\Delta }}{\rm{\delta }}}_{{\rm{N}}}{/5)}^{{\rm{2}}}}$$

where ${{\rm{\Delta }}{\rm{\delta }}}_{{\rm{HN}}}$ and ${{\rm{\Delta }}{\rm{\delta }}}_{{\rm{N}}}$ are the observed chemical shift differences in the backbone amide ^1^H and ^15^N spins, respectively, between the wild type and the mutant. The perturbation of backbone amide chemical shifts for most residues due to the mutation of the tryptophan residue at 514^th^ position to an arginine was found to exhibit an average (±standard deviation) deviation of 0.073 (±0.085) ppm for all residues, for which the difference could be obtained. Only 11 residues had a change in amide chemical shift higher than 0.15 ppm including F451, I469, K470, R471, L479, Y481, F483, A499, A504, T505 and L530. The residue specific plot of the amide chemical shift change (Fig. 4d) showed perturbations in the backbone amide chemical shifts after introducing the W514R mutation, with a majority of changes located in β strands 4, 5, 6 and loop between strands 6 and 7. This indicates the possibility of moderate structural changes in the mutant as compared to the wild type (Fig. 4b).

^13^C^α^ chemical shifts are known to be highly sensitive reporter of the protein’s secondary structure^40^ and hence, we compared the ^13^C^α^ chemical shifts changes due to W514R mutation to investigate secondary structural changes associated with it. Figure 4c shows that ^13^C^α^ chemical shifts of the wild type and the mutant share a linear correlation with r^2^ = 0.998. We also calculated the residue specific backbone ^13^C^α^ chemical shift difference between the two proteins as ${\rm{\Delta }}{\delta }_{C{\rm{\alpha }}}={\delta }_{C{\rm{\alpha }}}({\rm{wild}}\,{\rm{type}})-\,{\delta }_{C{\rm{\alpha }}}({\rm{mutant}})\,$and the average (±standard deviation) of ${\rm{\Delta }}{\delta }_{C{\rm{\alpha }}}$ was found to be 0.021 (±0.207) ppm (Fig. 4d,e). These results taken together indicate a detectable perturbation to the global fold as a sequel to the mutation.

**Figure 5:**
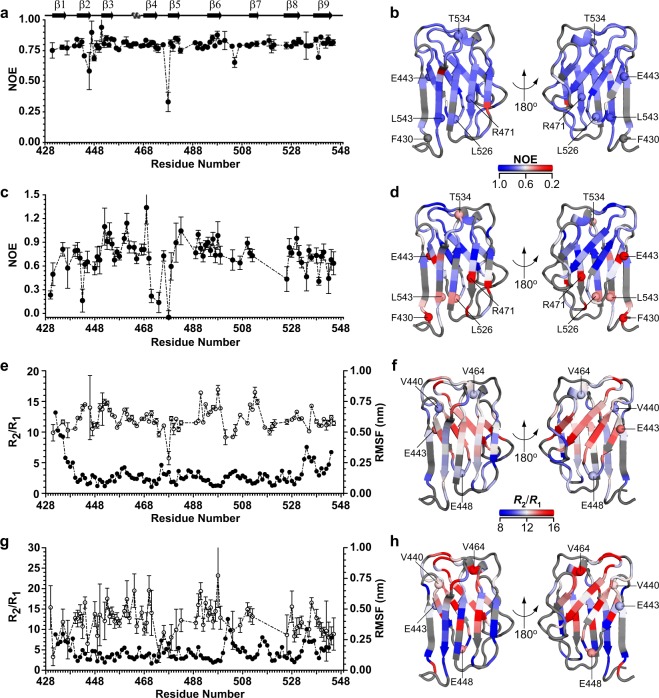
Ig W514R shows different dynamics compared to the wild type: Plots of residue specific {^1^H}-^15^N-heteronuclear NOE of wild type (**a**) and mutant Ig W514R (**c**) respectively, while (**b)** (wild type) and (**d)** (mutant) represent the corresponding values mapped on the NMR structure (PDB: 1IVT)^12^. Plots of *R*_2_/*R*_1_ relaxation ratios and the RMSF (root mean square fluctuation) from MD simulations of wild type (**e**) and mutant Ig W514R (**g**). *R*_2_/*R*_1_ relaxation ratios are mapped on the NMR structure as shown in (**f)** (wild type) and (**h)** (mutant) respectively. Some of the residues showing differences in their dynamic behaviour in wild type and mutant are represented as sphere in the NMR structures.

To probe changes in the backbone flexibility between the wild type and the mutant we measured their backbone ^15^N spin relaxation rates *R*_1_, *R*_2_ and NOE (Fig. 5a–d and Supplementary Fig. S3).The backbone motions of protein, including its global and local internal motions, modulate the relaxation behaviour, and hence, their relaxation rates, *R*_1_, *R*_2_ and {^1^H}-^15^N-NOE of ^15^N spins. It has been shown that an increase in the *R*_2_/*R*_1_ ratio for a stretch of residues in comparison to the neighbouring residues is likely to result from conformational exchange in the microsecond-millisecond timescale, while lower values of *R*_2_/*R*_1_ ratio indicates motions in the picoseconds – nanosecond timescale, likely as a result of local flexibility^41^. In our case, we found that for wild type protein, an average (±standard deviation) NOE of 0.793 (±0.07) indicates a fairly rigid backbone, though the residues in the vicinity of E444-E448 had a lower NOE value due to higher flexibility (Fig. 5a,b). This value is similar to the earlier report by Krimm *et al*.^39^. In comparison, the average NOE value for the mutant was 0.725 (±0.234), indicating an overall more dynamic backbone (Fig. 5c,d). The regions harbouring residues E444-E448 and R471-L479 were found to have increased dynamics in the mutant in comparison to the wild type, with more significant motion noted in the latter region for the mutant. The average values (±standard deviation) of *R*_1_ and*R*_2_, and *R*_2_*/R*_1_ were 1.682 (±0.282) Hz and 20.09 (±1.98) Hz for the wild type, and 1.950 (±0.784) Hz and 21.74 (±3.76) Hz for the mutant respectively. For residues where data were available for both *R*_1_ and *R*_2_, the average (±standard deviation) *R*_2_/*R*_1_ was 12.18 ± 1.77 Hz for the wild type. In contrast, the average of the *R*_2_/*R*_1_ ratio for the mutant was 12.22 ± 3.66 Hz. The results of *R*_2_/*R*_1_ ratio (Fig. 5e,f) showed that residues of the beta sheet formed by the β-strands 2, 3, 6 and 7 have higher *R*_2_/*R*_1_ ratio which is likely to be due to slow timescale (µs – ms) conformational exchange. While the mean values are similar for both proteins, the variation of *R*_2_/*R*_1_in mutant is significantly higher, indicating that the mutant undergoes elevated motions in the same regions as in the wild type but to larger extents (Fig. 5g,h). Not surprisingly, much of the fast conformational flexibility is localized in the loop regions between the β-strands 4–5 and extending well into the latter strand^42^. The rotational correlation time (*τ*_c_), which is proportional to the molecular weight, was ~10.7 ns for both the wild type and the mutant proteins. Given that the numerical values of rotational correlation times are found to be approximately half the effective molecular weight of proteins (at 25 °C), our observed values of *τ*_c_ for indicates that under the conditions of NMR experiments, both proteins exist as mixtures of predominantly monomers and higher oligomeric species The explanation becomes complicated by the fact that the exact distribution of the oligomers in solution for the mutant could not be ascertained.

**Figure 6:**
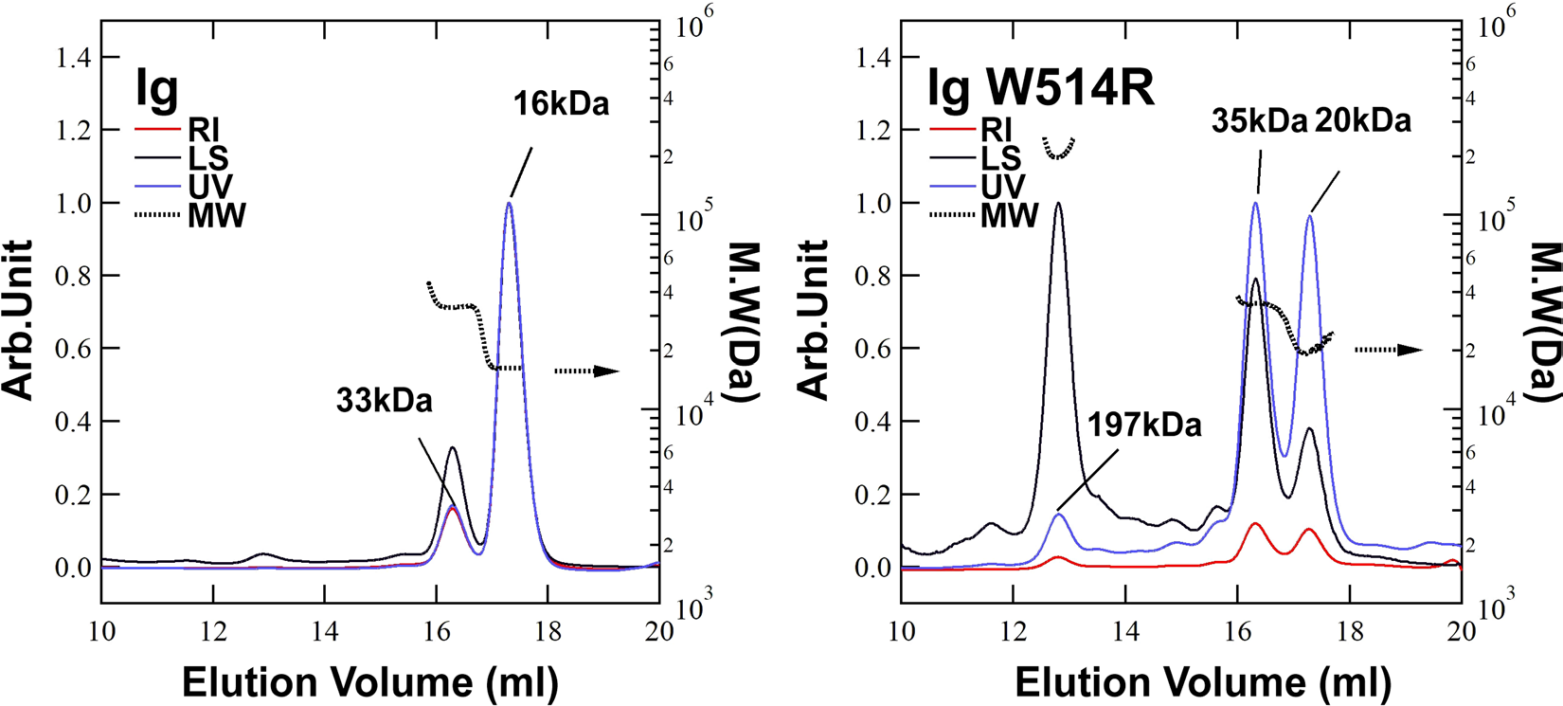
Ig W514R affects the state of association of Ig domain: SEC-MALS analysis of Ig and Ig W514R showing the different forms of the proteins respectively. (red line) Refractive index. (black line) Laser light scattering. (blue line) UV absorbance 280 nm. (black dot line) Molecular weight (right axis).

The possibility of existence of oligomers of Ig and Ig W514R, as suggested from NMR measurements of *τ*_c_ as well as fluorescence anisotropy, was checked by size exclusion chromatography-multiple angle light scattering (SEC-MALS) at 3 mg/ml concentration.The results showed that Ig exists predominantly as a monomer with minor contribution of the dimer (Fig. 6a) whereas the major components of Ig W514R are 35 kD and 197 kD entities (Fig. 6b). While 35 kD peak corresponds roughly to a dimer, the 197 kD peak corresponds to a 12-mer oligomeric species. This finding presents an explanation for an inherent instability of the mutant which is thus found to be susceptible to increased homooligomeric association thereby eliciting an out-of-phase property at high concentrations.

### Ig W514R unfolds at lower temperature

**Figure 7:**
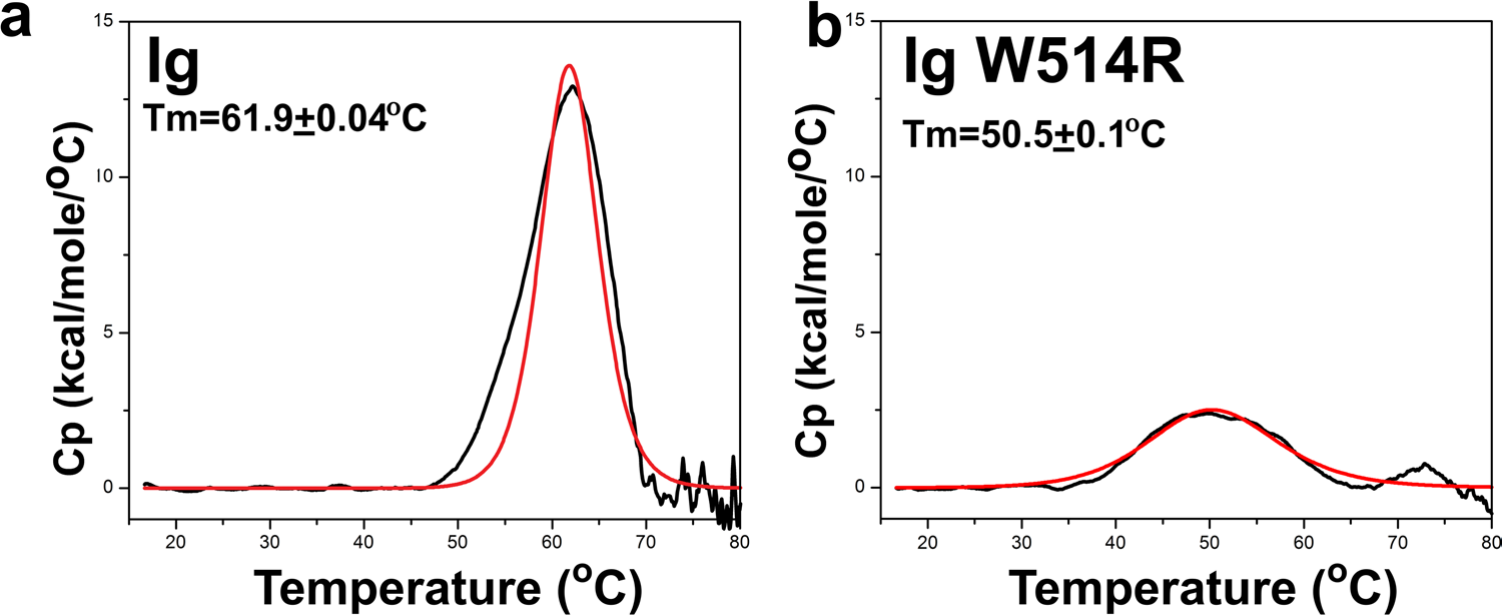
Wild type and mutant Ig unfold differently: Thermograms from differential scanning calorimetry to determine the thermal stability and corresponding unfolding behaviour of (**a**) Ig and (**b**) Ig W514R. Black and red represent experimental data and fitted data respectively.

Subsequently, in the logical flow of experiments, it is worthwhile to examine if the thermodynamic stability of the proteins are altered as a sequel to this oligomerization phenomenon. This prompted us to investigate the thermal unfolding behaviour of Ig W514R which is very likely to be affected by oligomerization tendencies. Therefore differential scanning calorimetry was used as a sensitive method to analyse heat capacity which is a signature of the thermal stability of a protein. We used 10 µM of each protein in PB. Ig exhibited a single transition point at 61.9 ± 0.04 °C (Fig. 7a) whereas Ig W514R unfolded at a lower temperature of 50.5 ± 0.1 °C (Fig. 7b) respectively. This is clearly due to different molecular state of associations of the two proteins.

Furthermore molar heat capacity (C_p_) values of Ig were almost 5 times that of Ig W514R. The calorimetric enthalpy (ΔH_cal_) was calculated by integrating the area under the thermograms which can be elucidated by the following equation:

$${\rm{\Delta }}{H}_{cal}=\int {\rm{\Delta }}{C}_{p}dT$$

where ΔH_cal_ & ΔC_p_ are calorimetric enthalpy and change in molar heat capacity respectively.

It can thus be concluded from the thermograms that both the proteins unfolded via a two-state process by absorbing heat. ΔH (observed) values were found out to be 110.1 ± 0.6 kcal/mol and 45.6 ± 0.3 kcal/mol for Ig and Ig W514R respectively. Thus, the unfolding of the mutant protein appears to be a co-operative process alongside with the dissociation of the oligomers which happens on increasing the temperature. Furthermore, a careful investigation of the mutant thermograms reveals a relatively broad peak which comes from a distribution of multiple forms of oligomers.

### Computational analysis shows a differential equilibrium dynamics in Ig W514R

**Figure 8:**
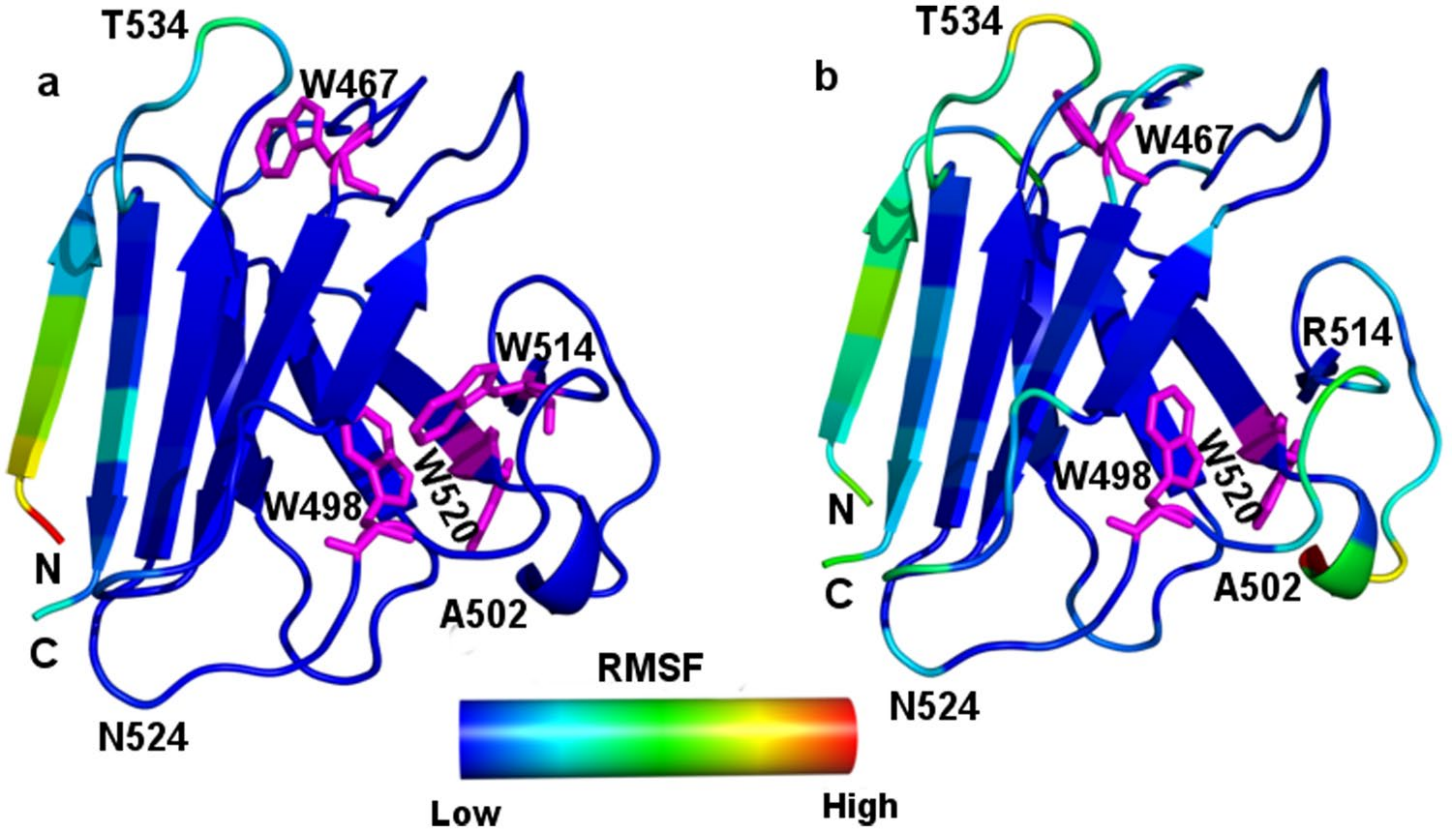
Average structures of Ig W514R fluctuates from Ig: RMSF values of amino acids (nm) from the MD simulation are mapped on the average structures of (**a**) Ig and (**b**) Ig W514R.Tryptohan residues are shown as stick model with magenta colour.

Subsequent root mean square deviation (RMSD) analysis elucidated that Ig was stabilized with an average RMSD of 0.25 nm within 25 ns. The C^α^RMSD of the IgW514R however was stabilized after 150 ns of simulation, with a higher average RMSD of ~0.55 nm as compared to the energy minimized IgW514R structure. It indicates that upon mutation from the hydrophobic residue W at position 514 to hydrophilic charged residue R, the protein structure required more time to attain equilibrium state, as expected. We thus computed the equilibrated trajectories for the last 100 ns simulation time in both cases. Mapping of root mean square fluctuation (RMSF) values (Fig. 8a,b) from the MD simulations over the averaged structure showed more flexibility as compared to those of the Ig W514R. Interestingly, the mutated residue W514R did not show much flexibility in the last 100 ns. The average radius of gyration (Supplementary Fig. S4) was found to be less in Ig (~1.33 nm) when compared to Ig W514R (~1.39 nm) which corresponds to compact and more voluminous conformations, respectively.

In order to understand the nature of the fast movements of some of the residues in Ig W514R, we have carried out principal component analysis. Most of the motions (~75%) were found to be captured by the first eigen value in both the cases. Porcupine analysis of the projected vectors further clarified directions of the motions in Ig and Ig W514R. Porcupine plot analysis showed that the direction of motion in the Ig W514R varied than Ig. In Ig protein significant motions were observed in N and C terminal regions only, whereas Ig W514R showed high intensity motions along the plane in the direction of the residues R500 to T510, which lies between β4-β5 strands. In addition, another significant motion was observed in Ig W514R between residues K515 to W520, which lies in between β6-β7 stands. Direction of this motion for these residues towards T480 and Y481 of the β5 strand indicates that the mutation had resulted in the altered motion in Ig W514R protein (Supplementary Fig. S5). We analyzed the surface exposure of the residues from trajectories. Most of them showed similar solvent accessible surface area(SASA) values, except W467, which became significantly more buried in Ig W514R mutated structure (~0.34 nm^2^ in the mutant Ig W514R compared to ~0.49 nm^2^ in the native Ig). This is in good accordance with our experimental data.

The MD simulations also elucidated changes in the secondary structural elements during the simulations upon mutation (Supplementary Fig. S6). Some of which are highlighted as important and noticeable changes. We observed a conformational change from bend to coil conformation near the tryptophan regions around W467 and W520, whereas in W498 interrupted β-sheet conformation was observed in Ig W514R protein, which was otherwise continuous in Ig. In Ig, the residues between positions A500-T505 appeared as turn and coil conformations till the end of the simulation but in Ig W514R, this region showed bend and turns with more traces of β-bridge conformation. In addition we also observed the structural perturbations around the mutated region between A516-G521. In this region Ig showed a bend conformation, whereas for Ig W514R was a bend to coil conformational flow.

### Impact of Mutation on Electrostatic Surface of the protein systems

**Figure 9:**
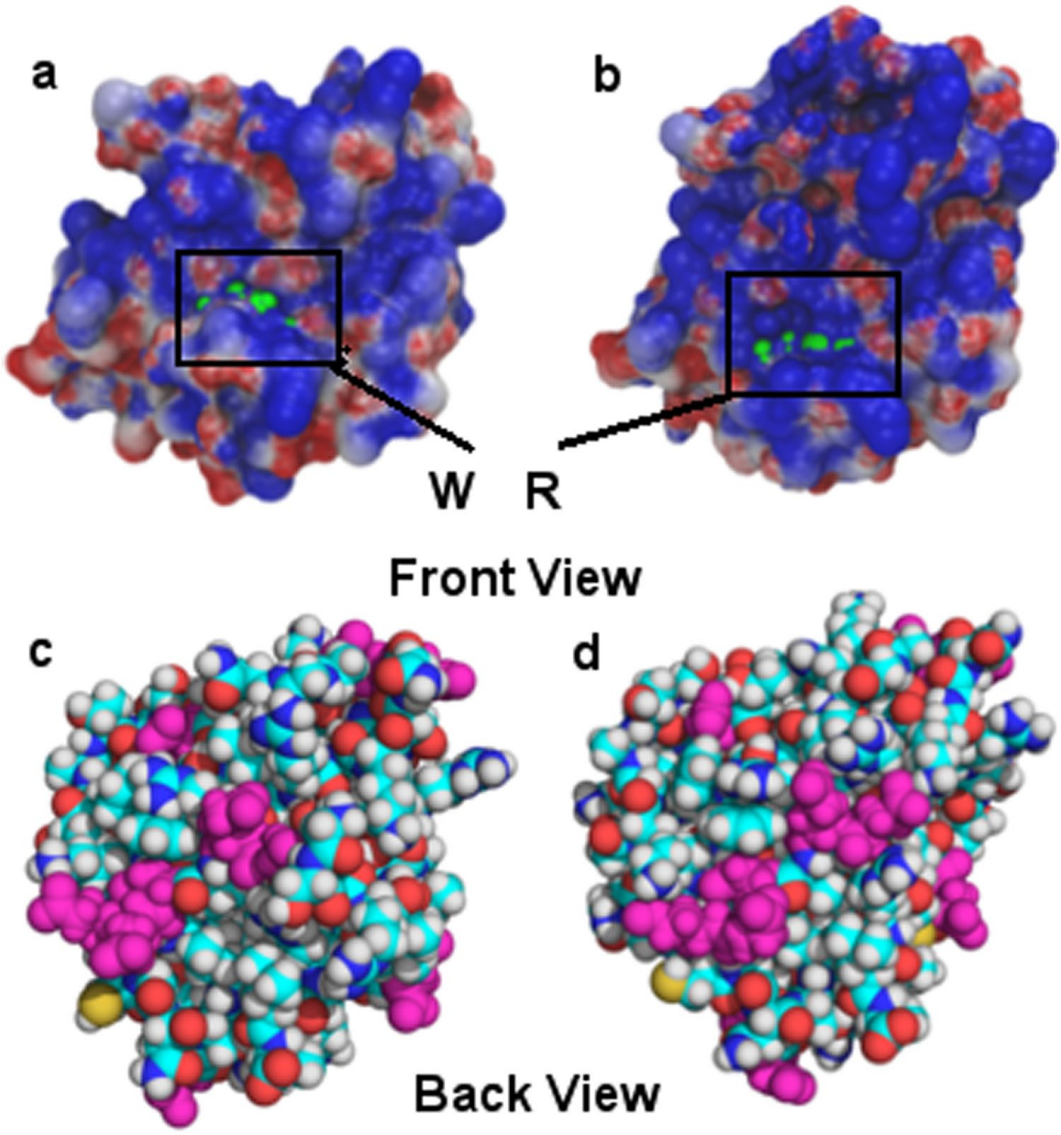
Electrostatic potential map for Ig and Ig W514R proteins were obtained using an APBS tool: The negative electrostatic potential is highlighted in red, and the positive potential is highlighted in blue. Green color marking is for mutated residue for (**a**) Ig and (**b**) Ig W514R. The view of the opposite face as space filled model with Asp and Glu residues highlighted in pink colour for (**c**) Ig and (**d**) Ig W514R.

It is well known that electrostatic forces and energies are essential for the interactions between macromolecular^43–45^. It may also be significant for our study because C-terminal domain of lamin A/C has been ascribed to be a site of interaction with many nuclear proteins. In the present study, we found that the electrostatic surface potential fluctuated between Ig and Ig W514R proteins at the mutated region (Fig. 9) at 300 ns. It showed that near the mutated region, the surface of Ig W514R protein displayed increased positive electrostatic potentials as compared to native Ig protein, whereas the distribution of negatively charged residues on the opposite faces showed minor differences (Fig. 9c,d). Therefore, the positive face of Ig W514R might exhibit a stronger propensity to bind to the opposite face of another molecule of Ig W514R with increased affinity. This nicely corroboration to the elevated aggregation behaviour of the protein from our *in vitro* and *ex vivo* results discussed in earlier sections.

### W514R mutant alters laminar morphology drastically

**Figure 10:**
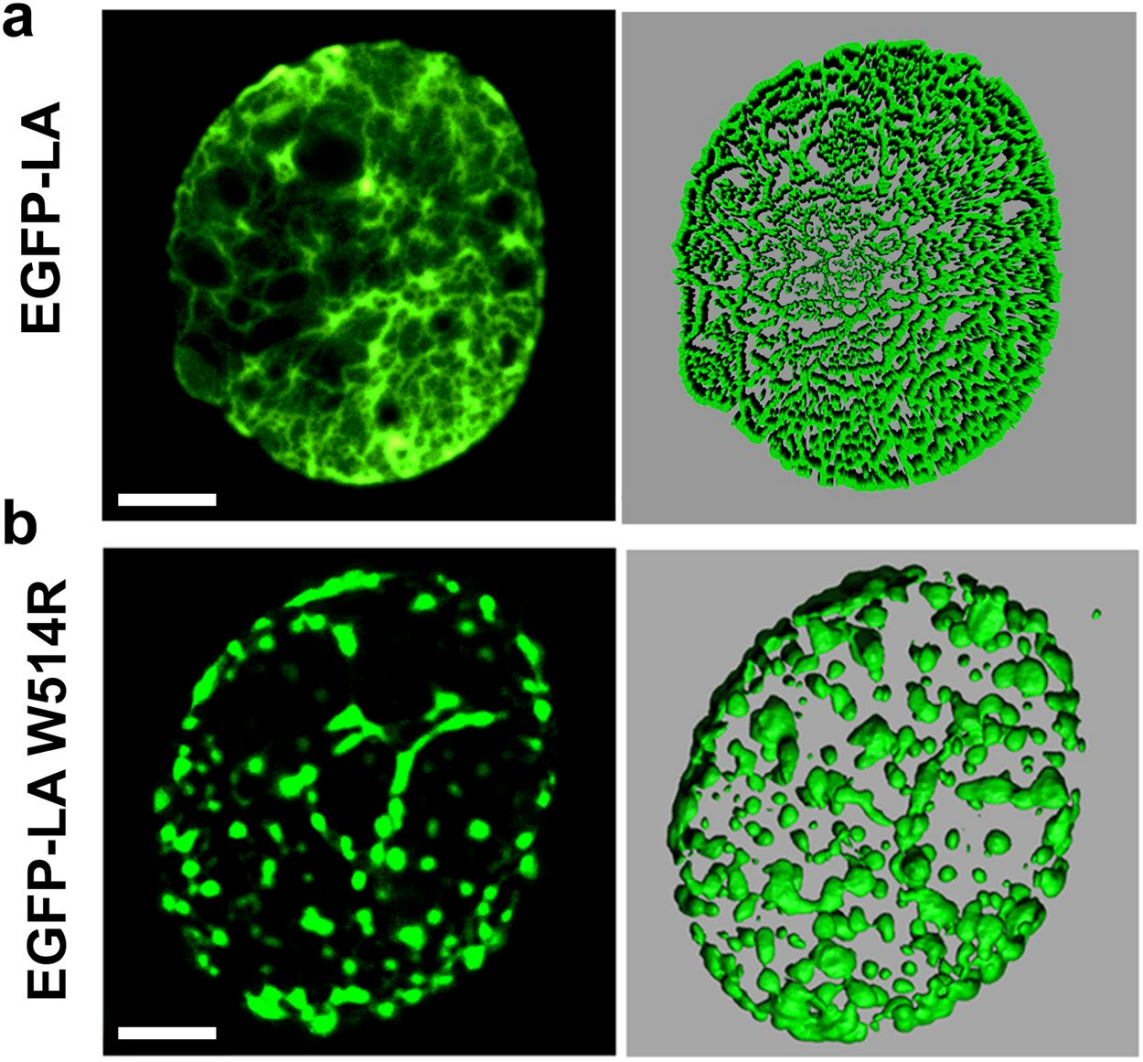
W514R overexpression in C2C12 leads to increased mesh size: (**a**) Panels show a single plane and 3D surface view of EGFP-LA and EGFP-LA W514R. Scale bar = 5 μm.

**Figure 11:**
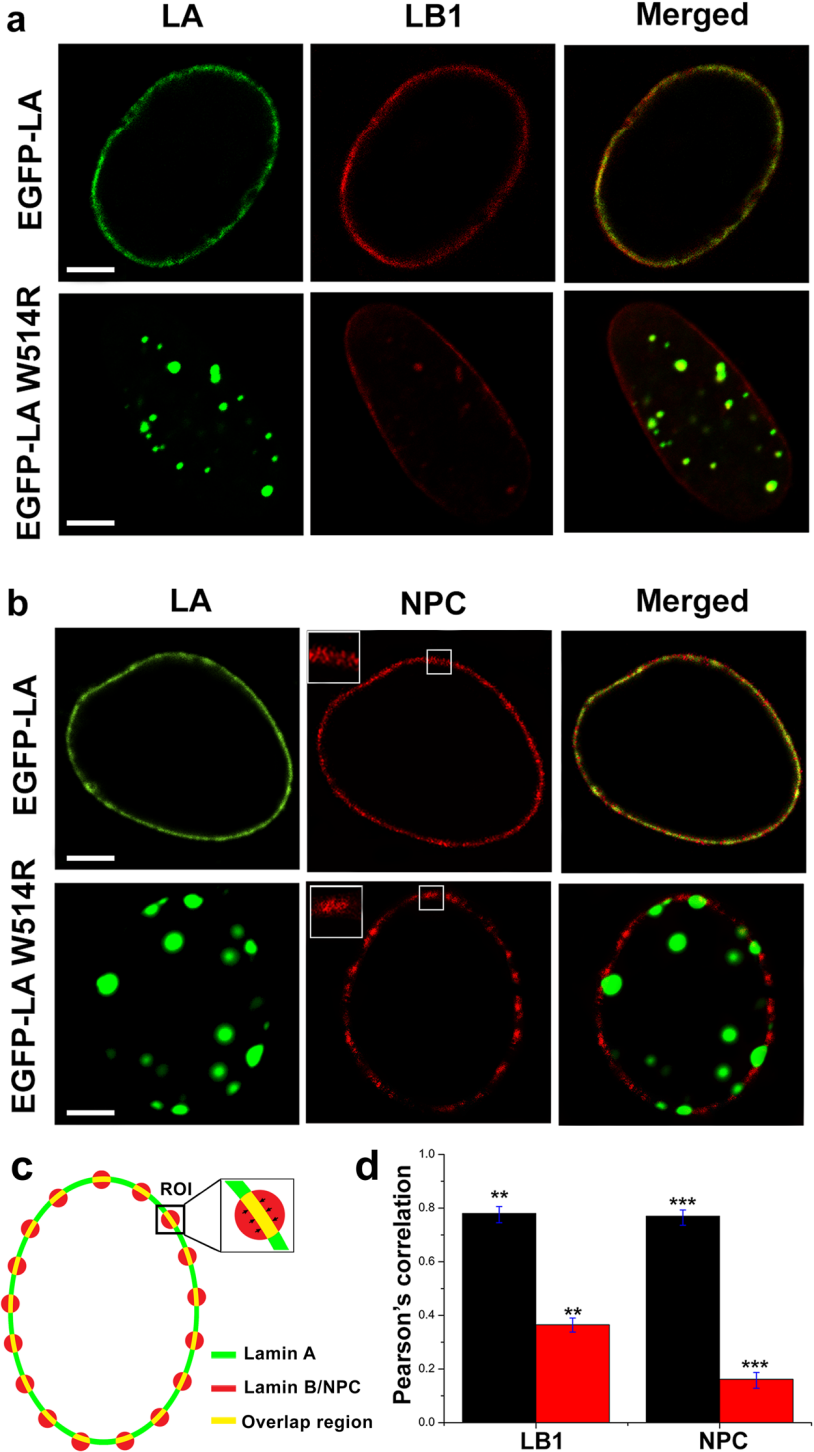
W514R alters the normal distribution pattern of nuclear envelop associated nuclear pore complex (NPC): (**a**) Distribution of LB1 on nuclear lamina shown in a single plain of wild type and mutant transfected cells. (**b**) Localization of nuclear pore complex on the nuclear rim has been elucidated in the two panels for wild type and mutant transfected cells. Insets marked by white squares represent zoomed (~6 fold) portion of the rim depicted by square shaped boxes. Scale bar = 5 μm. (**c**) Schematic illustration to calculate pearson correlation of selected region of interest (ROIs) on the periphery of nucleus through NIS Elements version 4.13.00 (build 914). (**d**) Pearson correlation for the co-localization of LA with LB1 & Nuclear pore complex. Black bar represents the wild type and red bar represents the mutant W514R (Error bars represent s.d., **represents p < 0.001, ***represents p < 0.0001).

Till now we have shown the deleterious effects of the mutation on the structure and the dynamics of the Ig domain in isolation. These findings led us to wonder if W514R induces any abnormality in the nuclear architecture. So, we transfected C2C12 cell lines with EGFP-tagged wild type (EGFP-LA) or mutant LMNA (EGFP-LA W514R) constructs. Similar studies in C2C12 cell lines were reported by Håkelien *et al*. for another myodystrophic mutant R453W^46^. It is emphasized here that we used full length lamin A proteins with EGFP reporter as Ig fold domain alone is incapable of entering the nucleus and is biologically irrelevant. Wild type lamin A protein showed uniform meshwork like structure throughout the nucleus whereas the mutant W514R showed disrupted network with visible aggregates distributed throughout the nuclei in almost 60–70% of the cases as shown in Fig. 10. We have shown the validity of our statement in representative fields of view as shown in Fig. S2. Approximately 200 nuclei for each of wild type and mutant were analysed and a representative figure has been presented in Fig. 10 for clarity. This is similar to previous observations from our and other groups involving laminopathic mutants^16,47–49^. Our observation clarified that there was no change in form factor due to mutation and no blebbing or herniation was observed as a result of over expressing this mutant. In order to rule out any artefacts due to overexpression, the expression levels of the wild type and mutant were compared in blots (Supplementary Fig. S1), which was found to be similar. However, mesh size of the mutant network was significantly larger than that of the wild type as shown in Fig. 10. As the Ig fold domain of lamin A interacts with numerous proteins which are responsible in maintaining the structure of the nuclear envelope^37^, we decided to check the effect on the localization of lamin B1 and nuclear pore complex proteins present in the nuclear envelope. The distribution of lamin B1 coincided with that of EGFP-LA whereas NPC staining showed a clustering instead of uniform distribution on the nuclear envelope and also sequestered in the lamin A foci (Fig. 11a,b). The schematic of quantification (Fig. 11c) and extent of overlap has been shown as Pearson’s Correlation graph (Fig. 11d).

## Discussion

Substituting Trp by Arg at residue 514 results in a missense mutation leading to severe phenotypes of skeletal muscle dystrophy^38^. Dialynas *et al*. showed structural perturbations in mutants G449V and W514R primarily by HSQC-TROSY experiments. These mutants are reported to cause severe phenotypes in patients. Their results showed the presence of cytoplasmic aggregates of lamin and other nuclear proteins in Drosophila muscles and suggested a possible link of the lamins with Nrf2 signalling cascade and maintenance of redox homeostasis. Skeletal muscle dystrophy manifests itself with cardiac dysfunction abnormal gait, muscular atrophy with associated muscle weakness. The severity of the symptoms can extend into diagnostic regimes like defects in joint movement and contractures, muscle pain and wasting^50^.

We embarked upon a project to investigate the detailed structural perturbations of Ig-fold domain as a sequel to a mutation like W514R that causes skeletal muscle dystrophy. Initial cues from CD and fluorescence spectroscopy hinted at changes of secondary and tertiary structure of Ig-fold which prompted us to investigate more into details at the level of single amino acid residues. In retrospect, detailed thermostability analysis for the mutants R453W, R482Q/L/W were reported by Krimm *et al*. where denaturation temperature for the wild type domain was registered to be 62 °C. This is similar to our results obtained from differential scanning calorimetry. R453W denatured at 43 °C while R482Q/W showed utmost resemblance to the wild type Ig fold^39^. We extended these findings to NMR spin relaxation experiments and chemical shift perturbation analysis which have not been hitherto performed for the mutant W514R. We could thus elaborate in this work, that W514R induces increased oligomerization propensity in the protein which could result in cytoplasmic aggregates as mentioned by Dialynas *et al*. Our result robustly calculated the Rg from MD simulations and showed that the mutant Ig W514R has bigger Rg compared to Ig. Interestingly, Qin *et al*. working on a similar approach, performed replica exchange molecular dynamics simulations on the tail domain of the wild type and LAΔ50 and calculated end-to-end length of the domain which depicted that the LAΔ50 tail domain is more compact than its wild type counterpart. This data was further verified by thermal denaturation profiles as well as comparing hydrodynamic radius of the two proteins^51^. Our MD simulation data nicely agreed with the difference in NOE between Ig and Ig W514R. Nevertheless, we found an explanation in the change of surface potential to account for its increased tendency to self associate up to 12-mer as obtained by SEC-MALS analysis. The fundamental observed differences in biophysical parameters between the native/wild type and W514R have been summarized in the Table 1. At the cellular level, the increased oligomerization behaviour of Ig W514R manifested itself in the form of misshapen laminar network. Interestingly, the lamin B1 network was not seen to be altered. This is in good agreement with the earlier data from Dialynas *et al*.^38^ which showed no changes in nuclear strain in drosophila muscles. Nonetheless, a gross abnormality in the distribution of nuclear pore complex (NPC) is a novel observation from our results, which further tells us that the molecular interactions mediated by the Ig fold domain are perturbed due to this mutation. This could point to a probable defect in nuclear transport mechanism. This aspect has not been shown by Dialynas *et al*.^38^ and this could offer yet another angle in explaining the pathobiology of muscular dystrophy. In retrospect, the interaction of Nup 88 with Ig fold mutants R453W & R482Q were elucidated to be impaired^28^. Similarly Nup 153 was also shown to bind to the Ig fold domain of A and B-type lamins and affected in a similar fashion in the case of the mutants R482Q and R453W^29^. This lamina-Nup153 interaction has greater implication in transcription regulation^52,53^.

**Table 1:** Summary of comparative analyses between Ig and Ig W514R.

Proteins	Anisotropy	Transition Temp (°C)	ΔH (kcal/mole)	NOE (avg)	Avg Rg (nm)
Ig	0.09	61.9 ± 0.04	110.1 ± 0.6	0.793 ± 0.07	1.33
Ig W514R	0.19	50.5 ± 0.1	45.6 ± 0.3	0.725 ± 0.234	1.30

Any structural and dynamical changes in the laminar structure are extremely likely to abrogate proper mechanotransduction phenomenon which encompasses transmission of external mechanical forces to the nucleus via LINC complex proteins. Mechanical stimuli applied to isolated nuclei buckle them easily^54,55^. Such improper mechanotransduction produces severity in the form of skeletal muscle dystrophy. Moreover MD simulations revealed wild type and the mutant proteins differ appreciably in the local dynamics thereby pointing to differential flexibility of the proteins as observed from porcupine plot. Taken together, this is the first report of its kind, where the impact of a laminopathic mutation on the structure and dynamics of the protein has been elucidated *in vitro*. This can lead to a new perspective of understanding the different interactions of nuclear proteins with the Ig fold domain. Future studies of protein-protein interaction can be undertaken along this line, which can provide valuable insights into understanding the pathogenesis of a disease with respect to key signalling molecules.

## Methods

### Cloning, expression, and purification

Human lamin A Ig (428–552) was cloned into pQE-80L vector (Qiagen) using forward primer 5′-CGCGGATCCGCGAGCAGCTTCTCACAGCACGCACGCACTAGC-3′ containing Bam HI restriction site and reverse primer 5′CGGGGTACCCCGTTAGGAAGATCTTCCGTCCTCAACCACAGTCACTGAGCG-3′ containing a tandem BglII, a stop codon, Kpn 1 sites respectively as described by Bera *et al*.^26^. After amplification PCR products were digested by BamH1-HF and Kpn1-HF(New England Biolabs,USA) using manufacturer’s protocol. The PCR product was purified from 1% Agarose byGel Extraction Kit (Qiagen,Germany). Digested vector and PCR product were ligated using T4 DNA Ligase(NEB) at 16 °C for overnight. Following ligation the ligated product was transformed into XL1 blue for propagation and amplification of plasmid. Ig W514R was generated using site directed mutagenesis as described by Bhattacharjee *et al*.^47^ using Site Directed Mutagenesis Kit (Stratagene) and the following sense and antisense primers respectively 5′CTAACCGACCTGGTGAGGAAGGCACAGAAC-3′ and 5′ GTTCTGTGCCTTCCTCACCAGGTCGGTAG-3′. Correct clone for plasmid was selected based on colony PCR and Sanger sequencing. The plasmid thus generated was transformed into *E. coli* BL21DE3pLysS cells and protein expression was induced with 1 mM IPTG(AMRESCO) at an O.D_600_ of 0.5 up to 6 h at 37 °C. For NMR experiments cells were grown in M9 minimal medium as described earlier^56^. Cell pellets were lysed in 25 mM Tris-HCl (pH8.5), 250 mM NaCl, 1% Triton X-100 and purified to near homogeneity on a His-Trap column (GE Healthcare Biosciences, USA). Protein stocks for all subsequent experiments were finally dialyzed against 25 mM Tris-HCl (pH8.5), 250 mM NaCl to remove any residual imidazole that was used for elution. This is the final buffer and will be referred to as Protein Buffer (PB). Proteins were routinely analyzed on 4–20% gradient SDS-polyacrylamide gels unless otherwise mentioned.

### Western Blot

Expression of Ig proteins were verified by blotting with anti-His antibody (gift from Dr. Oishee Chakrabarti, SINP, Kolkata) at a dilution of 1:1500 in TBS-Tween (0.5%)−5%NFDM and a secondary antibody dilution of 1:1000 as described earlier^26^. EGFP tagged proteins were probed with anti-EGFP antibody (ab 184601) at a dilution of 1:1000.

### Circular Dichroism (CD)

Far-UV (200–250 nm) CD spectra of Ig and Ig W514R were recorded in Biologic MOS-450 (France) CD spectrometer with a quartz cuvette (Hellma) of path length 1 cm at a temperature of 25 °C to compare the secondary structure of the wild type and mutant proteins. 10 µM protein was used in each case. Before each experiment the protein samples were centrifuged at 13000 rpm for 20 min at 25 °C and the concentration was checked.

### Steady State Fluorescence Spectroscopy

Steady state fluorescence spectra and anisotropy of wild type and mutant Ig proteins were recorded in a Perkin-Elmer LS 55 luminescence spectrometer at an excitation wavelength of 295 nm and using quartz cuvettes (Hellma) with a path length of 1 cm. For both excitation and emission, slit widths were kept at 5 nm and temperature was maintained at 25° C. The absorbance values of proteins at 340 nm were in the range of 0.01–0.03. It thus eliminated the necessity for correction due to inner filter effects.

### Nuclear magnetic resonance (NMR) spectroscopy and data processing

For NMR experiments, 25 mM Tris-HCl (pH8.5), 250 mM NaCl buffer system was used. The NMR experiments were carried out at 25 °C with uniformly ^13^C, ^15^N-labelled wild type protein with a concentration of ~0.54 mM, packed in a volume of 300 μL in Shigemi microcell. Due to its high instability, NMR experiments of the mutant W514R were carried out at 20 °C. with two samples- ^13^C, ^15^N-labelled and ^15^N-labelled at the concentrations ~0.135 mM and ~0.150 μM, respectively. All NMR experiments were performed on Bruker Avance 600 MHz (14.1 T field) spectrometer with 5 mm TXI room temperature probe equipped with z-axis gradient. The {^15^N-^1^H} heteronuclear single quantum coherence (HSQC) spectrum of the wild type was partially assigned by reconstructing a pseudo-spectrum using the chemical shift availed from BMRB (entry number 5224)^57^. Subsequently, a suite of 3D triple resonance experiments, HNCA and HN(CA)CB, were acquired to verify these assignments as well as assign all the ambiguous assignments of the spectrum of the wild type protein^58^. {^15^N-^1^H} HSQC spectrum of the mutant protein was assigned by recording a 3D HNCA experiment using the same pulse schemes. To estimate the fast timescale dynamics at picosecond nanosecond range, residue specific backbone amide ^15^N longitudinal (*R*_1_) and transverse (*R*_2_) relaxation rates as well as {^1^H}- ^15^N heteronuclear nuclear Overhauser enhancements (NOEs) were recorded using the pulse sequences developed by Kay and co-workers^59^ and described in details in prior work^60^. Briefly, in case of the wild type, the *R*_1_ relaxation measurements were done employing relaxation delays 5, 65, 145, 245, 365, 525, 750 and 1200 ms, while the *R*_2_ relaxation measurements was done with delays 8.5, 17.0, 25.4, 34.0, 50.9, 68.0, 84.8 and 101.8 ms. On the other hand the *R*_1_ relaxation measurements for the mutant were done employing relaxation delays of 5, 55, 155, 305, 605 and 1005 ms, while the *R*_2_ relaxation measurements were done using the delays 8.5, 17.0, 25.4, 42.4, 68.0 and 101.8 ms. {^1^H}- ^15^N NOE measurements for both the wild type and the mutant -were done by acquiring a reference spectrum with a 5 s relaxation delay and without any presaturation pulse, while the NOE spectrum was recorded with a 1 s relaxation delay and a 4 s presaturation delay. The pulse sequences used were furnished with gradient and sensitivity enhancement^61,62^, and the optimum water suppression was achieved by employing flip back and 3–9–19 binomial pulses^63^. Any potential sample heating was abolished by incorporating additional radio frequency (RF) pulses following the acquisition period, for experiments employing variable amount of high power RF irradiation periods as well as recording relaxation data in an interleaved manner^64^. All NMR data were processed with NMRPipe program^65^ and the spectral assignments were done using Sparky (http://www.cgl.ucsf.edu/home/sparky). A Lorentzian to Gaussian apodization window prior to Fourier transformation was used and the peak intensities wlere extracted successively after fitting the peaks to Gaussian line shapes using nLinLS sub-routine in NMRpipe. The residue specific ^15^N relaxation rate constants, *R*_1_ and *R*_2_, were extracted by fitting the trajectories of peak intensities plotted against relaxation delays to a single exponential function, while the ratio of cross-peak intensities with and without presatuaration pulses gave the site resolved {^1^H}- ^15^N NOEs. The root mean square (r.m.s.) spectral noise, estimated from NMRPipe, was used to calculate the errors in *R*_1_, *R*_2_ and NOE. An initial estimation of rotational correlation time was obtained from the ^15^N *R*_2_/*R*_1_ ratios^66^ using the program *r2r1_tm* (kindly provided by Dr. Arthur G. Palmer (Columbia University) {http://www.palmer.hs.columbia.edu/software.html}). The NMR structure of the protein (PDB: 1IVT)^19^ was appropriately rotated and translated using *pdbinertia* (http://www.palmer.hs.columbia.edu/software.html) and was introduced into the *r2r1_diffusion* (http://www.palmer.hs.columbia.edu/software.html), a program based on the method developed by Tjandra *et al*.^42^, along with the rotational correlation time estimated above. In order to avoid the influence of internal motions to the overall molecular tumbling, residues with *R*_2_/*R*_1_ ratio one standard deviation higher and lower than the average value were considered to have local dynamics and were excluded in *r2r1_diffusion* along with the residues with NOE value of one standard deviation less than the average value.

### Size exclusion chromatography-Multiple angle light scattering (SEC-MALS)

SEC-MALS measurements were performed with a Dawn Heleos II detector (Wyatt Technologies) at 20 °C. Samples were injected into a Superdex 200 increase 10/300 (GE healthcare). Injection volume of samples and flow rate were set to 30 µL and 0.6 mL/min, respectively. The size exclusion chromatography column was equilibrated in PB. These obtained data were analyzed by ASTRA 6 software (Wyatt Technologies).

### Differential scanning calorimetry (DSC)

Differential scanning calorimetry (DSC) was performed in VP-DSC Micro calorimeter (Microcal, LLC,Northampton, MA). Samples at a concentration of 40 µM were analyzed between 16° and 80° C for Ig and Ig W514R at a scan rate of 30° C/h. at approximately 30 psi pressure. Before each sample analysis, the instrument was thermally stabilized by at least 3 buffer scans. The buffer consisted of 25 mM Tris-HCl (pH 8.5), 250 mM NaCl. All the thermograms were analyzed by in-built VP Viewer software with origin 7.0.

### Molecular Dynamic (MD) simulations

Molecular dynamic simulations of Ig and IgW514R structures were performed using GROMOS96 53a6 force field^67^ with GROMACS 4.5.3 software^68^. The systems were solvated in a cubic box with explicit SPC model water molecules^69^. The distance between solute and box edge was at least 10 Å for each system. The systems were neutralized by adding Cl^−^ counter ions as necessary. Before MD simulations, the systems were energy minimized using the 10000-steps steepest descent followed by 1000000-steps conjugate gradient algorithms to remove all steric clashes. The equilibrated structures were subjected to production run of 300 ns. All the hydrogen bonds were constrained using LINCS algorithm^70^. Berendsen coupling^71^ was employed to maintain a constant temperature of 300 K with coupling constant of 0.1 ps and pressure was maintained at 1 bar with coupling constant of 0.5 ps. Periodic boundary condition was employed for defining ideal 3D tiling of the system. Long-range electrostatic interactions were calculated with the particle mesh Ewald method (PME)^72^. Short-range Coulombic and Lennard-Jones interactions distance cut-off was kept at 1.2 nm. The simulation trajectories were analyzed using various tools available in the GROMACS package. Principal component analysis (PCA) is used to identify the significant motions of proteins^73^. The C^α^ atomic motion of the top most eigenvector, which is the indicative of the fluctuations from the PCA analysis, was visualized using porcupine plots. To visualize the direction and extent of the principle motions of Ig and Ig W514R, porcupine plot analysis was performed using pymol^74^. Secondary Structure analysis for Ig and Ig W514R during MD simulations were carried out using definition of secondary structure of protein (DSSP) tool by Kabsch and Sander^75^.

### Electrostatic Potential Surface Calculation

Poisson- Boltzmann electrostatic potential was calculated for the last frames of the MD simulated structures of Ig and IgW514R using APBS tool^76^ and the surface electrostatic potential map was visualized using VMD^77^.

### Cell culture and transfection

C2C12 (myoblast) cell lines were cultured in Dulbecco’s modified Eagle’s medium (DMEM) from Himedia, supplemented with 10% fetal bovine serum (Gibco) and 5% penicillin/streptomycin (Sigma), and maintained in a humidified incubator at 37°C in 5% CO_2_. Transfection with pEGFP –human LA (EGFP- LA) and mutant LA (EGFP-LA W514R) were carried out with Lipofectamine 2000 (Invitrogen) according to manufacturer’s protocol at early passages (4–6) of 70% confluent cells and were kept in culture for 48 h post transfection. The transfection efficiency was quantified based on visualization of EGFP fluorescence in an inverted microscope.

### Indirect Immunofluorescence

For immunofluorescence, cells were plated on 18 mm/22 mm glass coverslips(Himedia) in 6 well culture dishes (nunc) one day before transfection. 48 hrs post transfection cells were washed with 1x PBS for 2 times 1 min each at room temperature. Followed by PBS washing cells were fixed with 4%Paraformaldehyde for 10 min at RT and permeabilized with 0.5% Triton X-100 for 5 min at Rt. Before and after cell fixation and permeabilization cells were washed with 1x PBS for 5 times 3 min each. Thereafter, cells were incubated with primary antibody (abcam anti-Lamin B1 antibody [ab16048] 1:500 dilution, abcam anti-nuclear pore complex proteins antibody [Mab 414] 1:1000 dilution for 2 hrs at RT. Followed by incubation with primary antibody cells were washed with PBS / 0.05% Tween 20 buffer and PBS 3 times at room temperature. Then cells were incubated with secondary antibody goat Anti-mouse IgG (H + L) secondary antibody, Alexa Fluor 594 conjugate Invitrogen (A11005) antibody for 1 hr at RT. Finally, cells were again washed with washing buffer and PBS 3 times each for 3 min at RT. Coverslips were mounted with Vectashield containing DAPI for staining DNA on glass slides (Himedia). At final stage, coverslips were sealed with transparent nail paint.

### Confocal Microscopy

Confocal images were captured in a NIKON Inverted Research ECLIPSE TiE Laser Scanning Confocal/NSIM Microscope. A Plan Apochromat VC 100x oil DIC N2 /1.40 135 NA/1.515 RI objective with an additional 4x digital zoom was used to visualize the nuclei. Images were captured with a Nikon A1RMP detector (Galvano mode). A multi-line Argon-Krypton laser (λex - 457/488/561 nm) was used at 3% of the original wattage (40 mW) for the green channel. A solid state laser (λex -561 nm) was used for the red channel at 5% of the original wattage (20 mW). A step size of 0.25 μm was maintained for capturing the Z stacks.

### Statistical Analysis for co-localization study

All calculations were done using Microcal Origin Pro 8. Student’s t-tests were performed to evaluate the significance of two sets of data which includes native and mutant proteins for n = 200. Two-tailed p values (95% confidence) were calculated. Error bars are standard deviation (s.d) as indicated in image.

## Electronic supplementary material

Supplementary information
