# Supplementary material for: Skeletal Muscle Dystrophy mutant of lamin A alters the structure and dynamics of the Ig fold domain
Source: Sci Rep. 2018 Sep 14;8:13793. doi: 10.1038/s41598-018-32227-2 (PMC6138676; doi:10.1038/s41598-018-32227-2)
Supplement: Supplementary file 1 — Supplementary information [file 41598_2018_32227_MOESM1_ESM.pdf]

Supporting Information

Skeletal Muscle Dystrophy mutant of lamin A alters the structure and dynamics of the Ig fold domain

Subarna Dutta*[1,4], Jitendra K Das*[2], Lakshmi Maganti[3], Maitree Bhattacharyya[4], Dhananjay Bhattacharyya[3], Sujoy Mukherjee[2]& Kaushik Sengupta[1]#

1. Biophysics & Structural Genomics Division, Saha Institute of Nuclear Physics, 1/AF Bidhan nagar, Kolkata-700064, West Bengal, India, Homi Bhabha National Institute
2. Structural Biology & Bio-Informatics Division, CSIR Indian Institute of Chemical Biology, 4, Raja S. C. Mullick Road,Kolkata-700032, West Bengal, India
3. Computational Science Division, Saha Institute of Nuclear Physics, 1/AF Bidhan nagar, Kolkata-700064, West Bengal, India, Homi Bhabha National Institute

4. Department of Biochemistry, University of Calcutta, 35 Ballygunge Circular Road, Kolkata-700019, West Bengal, India

#correspondence: kaushik.sengupta@saha.ac.in

*Authors contributed equally.

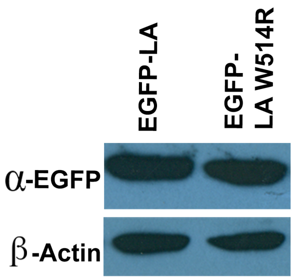

Supplementary Fig. S1: Immunoblot analyses from cell lysates of wild type and mutant LA transfected cells and probed with anti GFP antibody. β-actin was used as a loading control.

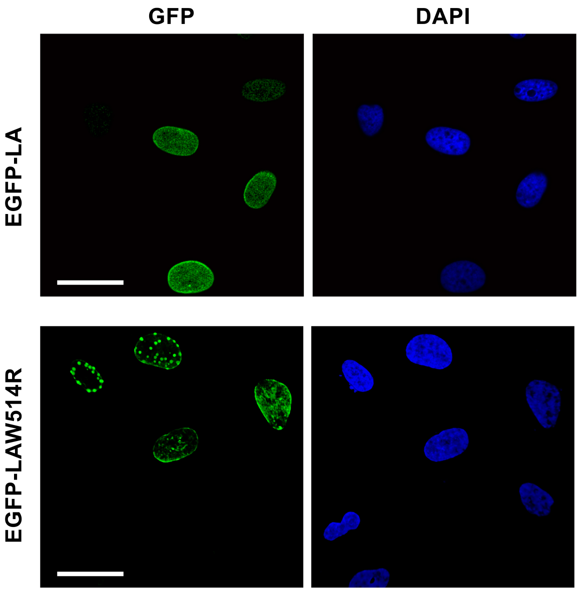

Supplementary Fig. S2. EGFP-LA and EGFP-LAW514R transfected nucleus of C2C12 cells under plan Apochromat VC 100x oil DIC N2 /1.40 135 NA/1.515 RI objective with an additional 1x digital zoom of NIKON Inverted Research ECLIPSE TiE Laser Scanning Confocal/NSIM Microscope. Scale bar = 10 µm.

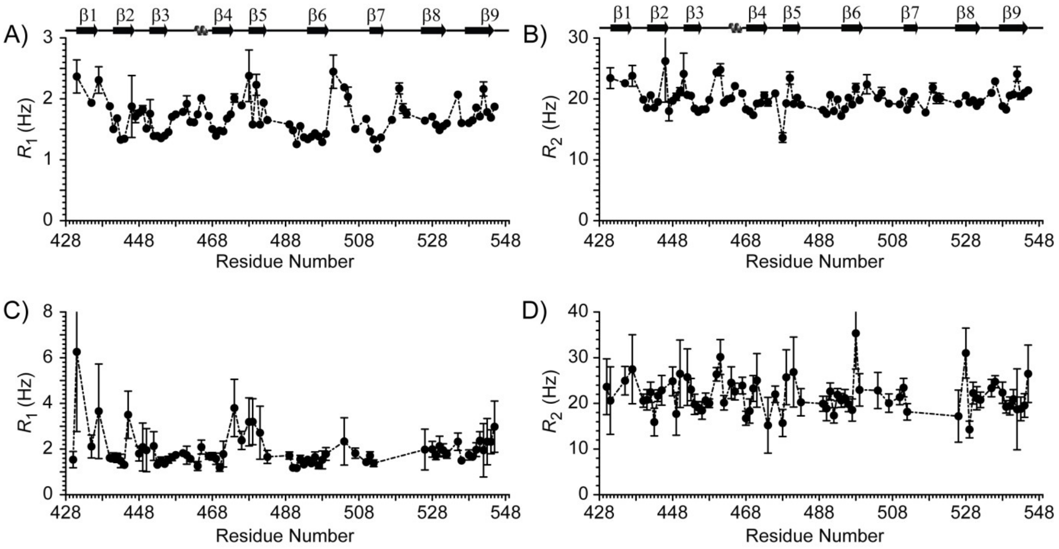

Supplementary Fig. S3: ^{15}N relaxation rates of Ig domain measured at 12.4 T external magnetic field. Plot of (**a**) longitudinal (R_1) and (**b**) transverse relaxation rates (R_2) of wild type Ig, respectively. Plot of (**c**) longitudinal (R_1) and (**d**) transverse relaxation rates (R_2) of mutant Ig W514R.

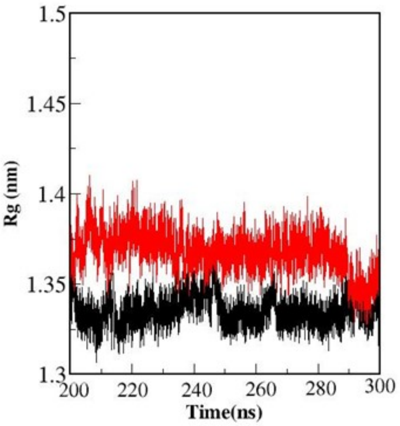

Supplementary Fig. S4: Radius of gyration of protein atoms during last 100 ns MD simulation time for Ig(black) and Ig W514R(red) proteins.

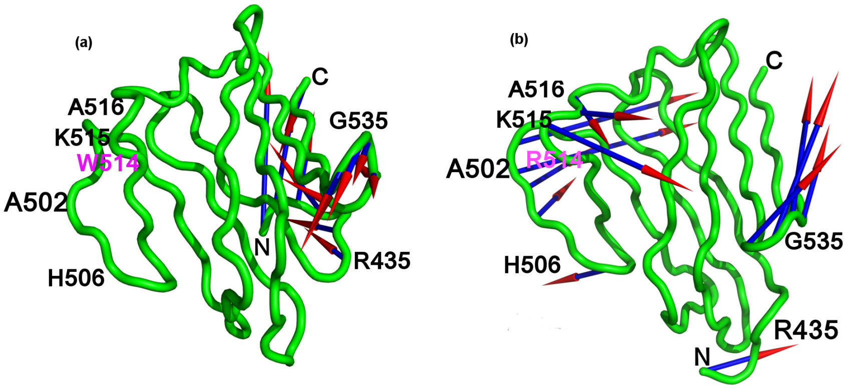

Supplementary Fig. S5: Porcupine plots of the first eigenvector for simulation of (a) Ig and (b) Ig W514R. The structures are shown as a backbone trace. The arrows attached to each Cα atom indicate the directions of the eigenvector and the size of each arrow shows the magnitudes of Cα atoms of the corresponding eigenvalue.

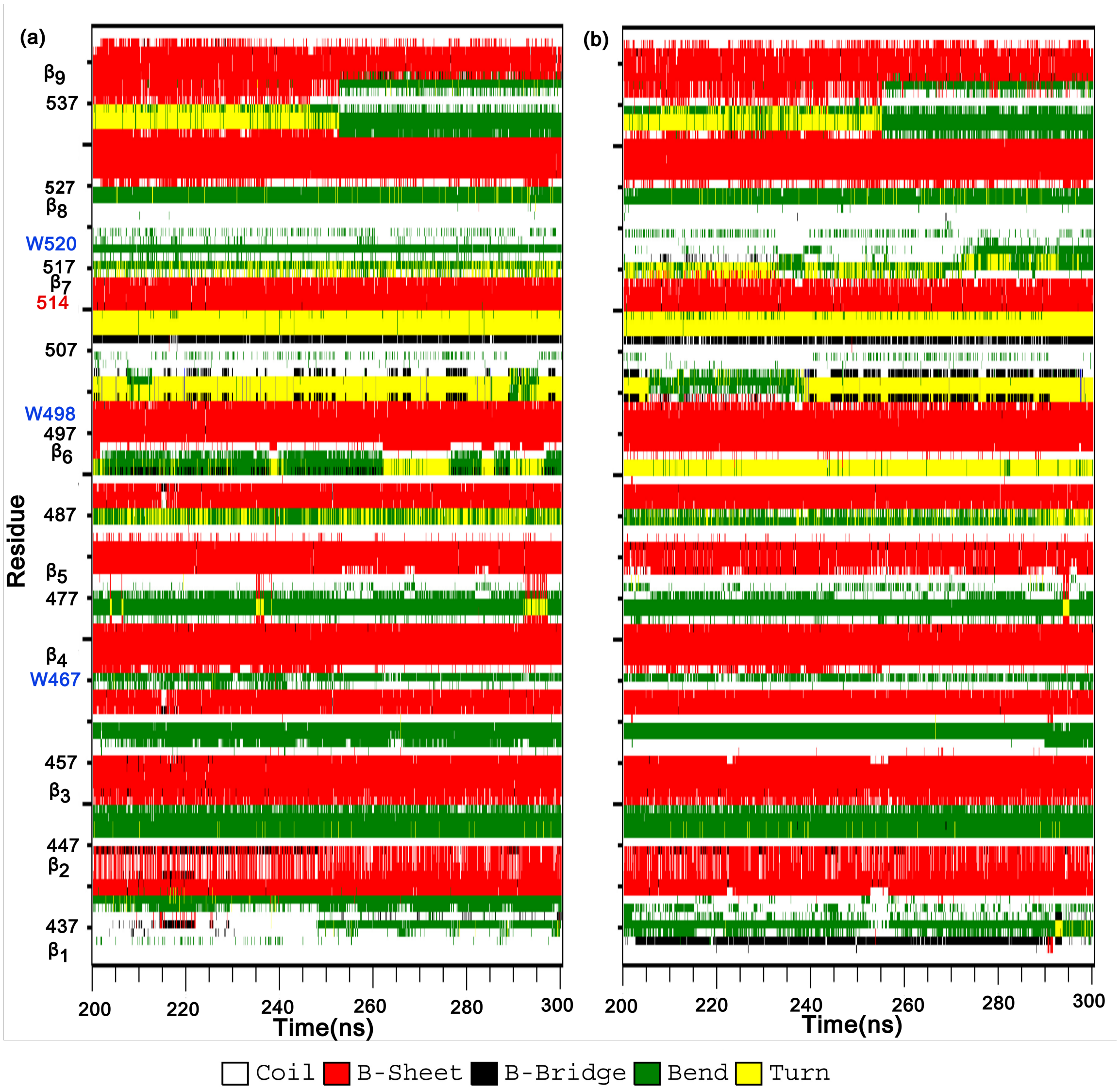

Supplementary Fig. S6. Secondary structures of protein residues (along Y-axis) at each time frame (along X-axis) during MD simulations as calculated by DSSP tool (a) Ig and (b) IgW514R proteins. Colour codes of each type of secondary structure are shown below.

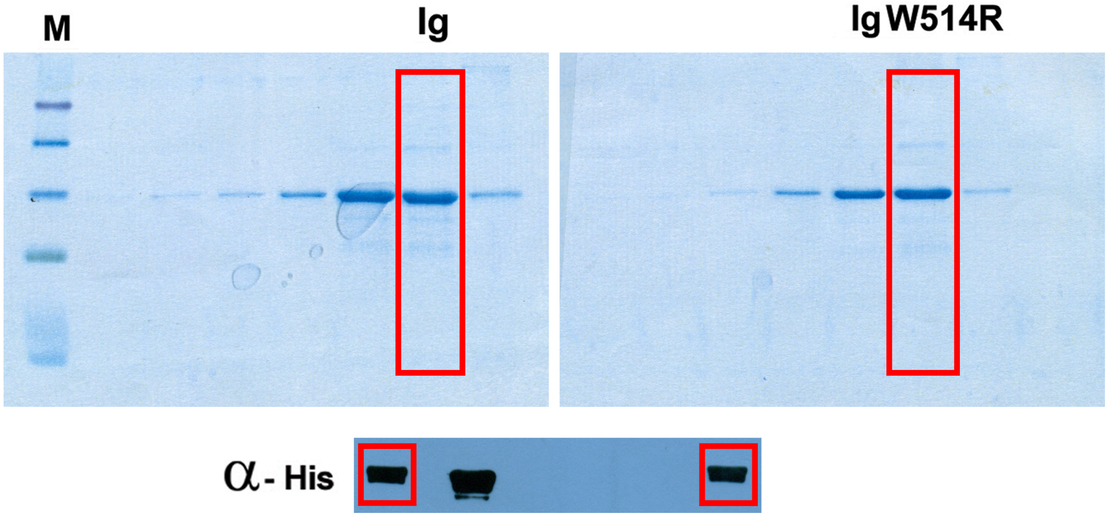

Supplementary Fig. S7. Analysis of purified proteins using 4-20% gradient gel and western blot of Ig and Ig W514R. Red boxes denote the cropped strips shown in Fig. 1.
